# Improving care of patients with low-risk differentiated thyroid carcinoma

**DOI:** 10.20945/2359-3997000000034

**Published:** 2018-03-23

**Affiliations:** 1 Universidade Federal do Rio de Janeiro Universidade Federal do Rio de Janeiro Hospital Universitário Clementino Fraga Filho Serviço de Endocrinologia Rio de Janeiro RJ Brasil Serviço de Endocrinologia do Hospital Universitário Clementino Fraga Filho, Universidade Federal do Rio de Janeiro (HUCFF/UFRJ), Rio de Janeiro, RJ, Brasil

In the last decade, imaging techniques had a great improvement in quality and became widely available all over the world for screening and diagnosis of the vast majority of malignancies. However, concerning thyroid nodule and cancer, we frequently face cases where micronodules were found incidentally by ultrasonography and other image modalities, often performed without clear indication. Some advocate this might be one of the main reasons for the rising incidence of thyroid nodules and microcarcinomas (1). Most Thyroid Societies advocate that nodules less than 0.5-1.0 cm should not be biopsied due to the low risk of those potential tumors (1). The proper utility of diagnostic tools, however, can be used to tailor the management of these nodules leading to a more accurate diagnosis and, therefore, a more adequate therapeutic approach (1).

In this issue of *Archives of Endocrinology and Metabolism* (AE&M), Macedo and cols. (2) described 195 thyroid nodules detected by ultrasound using two scoring systems: modified TI-RADS and ATA risk stratification, both scores based on ultrasonographic features. The results were compared to cytopathological analysis. Histopathological results, considered the golden standard for diagnosis of thyroid cancer, were available in 45 cases after surgery. They concluded that both TI-RADS and the ATA ultrasound guidelines have high sensitivity and NPV for the diagnosis of thyroid carcinoma, meaning they can accurately predict the probability of benignity. They also relate that both systems are easily applicable in a routine basis and can be used to tailor fine needle aspiration cytology (FNAC), avoiding unnecessary procedures (2). These results are in agreement with other studies that evaluated the ultrasound findings in order to define criteria for indication of FNAC in a suspicious nodules with accurate results.

Fama and cols. (3) provided further arguments for the better use of diagnostic tools to evaluate thyroid nodules. By retrospectively reporting a 12% histological incidence of papillary thyroid cancer (PTC) in 207 consecutive patients who, in a 1-year period, underwent thyroidectomy for benign multinodular goiter, they concluded that multinodular goiter can harbor preoperatively unsuspected PTCs, which may have already infiltrated the capsule and can be associated with PTC foci contralaterally, leading to the need of an adequate surgical approach (3). Therefore, once the option for surgery in a multinodular goiter is made, the correct assessment should be carried out in order to try to rule out the malignant nodules, helping the surgeon to avoid a higher rate of relapses and recurrences and to establish the correct surgical strategy.

Regarding treatment, it is already agreed that it should be individualized (4,5). The standardized treatment, which was used for all patients, is no longer applicable: total thyroidectomy followed by ablative radioiodine dose (4). All guidelines recommend that the first therapeutic decision for differentiated thyroid carcinomas should be based on risk factors, not only for rare cancers related death, but especially on the much more common risk of recurrence (4,5). For small tumors, accumulated experience of more than a decade mainly by Ito's group (6), recommends active surveillance for patients with microcarcinoma that meet very low risk criteria. In other centers, total thyroidectomy is indicated for almost all patients, and partial thyroidectomy may be performed in well-selected cases (7).

Regarding the indication of adjuvant radioiodine therapy after surgery, for most cases requiring further treatment, the use of 30 mCi has been proved to be as effective as 100 mCi, which has been indiscriminately advocated in the past for all patients (7). A detailed surgical report is essential in order to decide the best approach for each patient. Tumor histopathology is another important factor for the therapeutic decision. In the present issue of AE&M, Súss and cols. (8) showed that in properly selected low or even intermediate risk patients, follow-up without radioiodine indication is a plausible option.

More data on the tumor brings better decisions regarding the risks and benefits of radioiodine treatment (1). Pitoia and cols. (9) reviewed 210 DTC patients with low and intermediate risk of recurrence (RR) who underwent total thyroidectomy and remnant ablation, with 63 available historic pathologic samples (HPS). The RR and the response to therapy were first determinate by classic histological features (histological type, tumor size, capsular invasion, number of lymph node metastases) and, then, reassessed after observing additional histological features (vascular invasion, extrathyroidal extension, size of lymph node metastases, presence of extranodal extension, and/or status of the resection margins). A change in the RR category was observed in 16 of 63 cases (25.4%) A detailed report of specific features in the HPR of patients with DTC might give a more accurate RR classification and a better estimative of the response to treatment.

In conclusion, the management of such prevalent disease which leads to very low risk of death and recurrence should include the careful evaluation between risk and benefit of each approach, reassessed in every medical appointment with the proper indication of FNAC in the correct nodule, followed by the decision of the right surgery for each patient and also adjuvant therapy afterwards.
